# The use of herbal medicines during breastfeeding: a population-based survey in Western Australia

**DOI:** 10.1186/1472-6882-13-317

**Published:** 2013-11-13

**Authors:** Tin Fei Sim, Jillian Sherriff, H Laetitia Hattingh, Richard Parsons, Lisa BG Tee

**Affiliations:** 1 School of Pharmacy, Faculty of Health Sciences, Curtin University, Perth, Western Australia; 2 School of Public Health, Faculty of Health Sciences, Curtin University, Perth, Western Australia

**Keywords:** Herbal medicines, Breastfeeding, Lactation, Breastfeeding women, Survey, Prevalence

## Abstract

**Background:**

Main concerns for lactating women about medications include the safety of their breastfed infants and the potential effects of medication on quantity and quality of breast milk. While medicine treatments include conventional and complementary medicines, most studies to date have focused on evaluating the safety aspect of conventional medicines. Despite increasing popularity of herbal medicines, there are currently limited data available on the pattern of use and safety of these medicines during breastfeeding. This study aimed to identify the pattern of use of herbal medicines during breastfeeding in Perth, Western Australia, and to identify aspects which require further clinical research.

**Methods:**

This study was conducted using a self-administered questionnaire validated through two pilot studies. Participants were 18 years or older, breastfeeding or had breastfed in the past 12 months. Participants were recruited from various community and health centres, and through advertising in newspapers. Simple descriptive statistics were used to summarise the demographic profile and attitudes of respondents, using the SPSS statistical software.

**Results:**

A total of 304 questionnaires from eligible participants were returned (27.2% response rate) and analysed. Amongst the respondents, 59.9% took at least one herb for medicinal purposes during breastfeeding, whilst 24.3% reported the use of at least one herb to increase breast milk supply. Most commonly used herbs were fenugreek (18.4%), ginger (11.8%), dong quai (7.9%), chamomile (7.2%), garlic (6.6%) and blessed thistle (5.9%). The majority of participants (70.1%) believed that there was a lack of information resources, whilst 43.4% perceived herbal medicines to be safer than conventional medicines. Only 28.6% of users notified their doctor of their decision to use herbal medicine(s) during breastfeeding; 71.6% had previously refused or avoided conventional medicine treatments due to concerns regarding safety of their breastfed infants.

**Conclusions:**

The use of herbal medicines is common amongst breastfeeding women, while information supporting their safety and efficacy is lacking. This study has demonstrated the need for further research into commonly used herbal medicines. Evidence-based information should be available to breastfeeding women who wish to consider use of all medicines, including complementary medicines, to avoid unnecessary cessation of breastfeeding or compromising of pharmacotherapy.

## Background

Breastfeeding provides numerous benefits for newborn infants and mothers. Breast milk provides tailored nourishment to the growing needs of infants [1], offering optimal nutrition, improved cognitive performance and neurological development [2] and enhanced immunity [3,4]. It reduces the incidence of Sudden Infant Death Syndrome (SIDS), allergic/hypersensitivity diseases, and development of Type 1 (insulin dependent) and Type 2 (non-insulin dependent) diabetes mellitus [3,5-7] relative to the use of infant formula. Breastfeeding may also play a role in decreasing post-partum depression, bleeding, and improving weight control [8]. Furthermore, women who have a history of breastfeeding experience a reduced risk of osteoporosis and reduced incidence of breast and ovarian cancers [8-10]. Besides these health advantages, mothers and their babies are brought into closer contact through nursing itself [11]. Guideline 4 *“Encourage, support and promote breastfeeding”* of the Australian Dietary Guidelines 2013 published by the National Health and Medical Research Council [12] acknowledges the positive physical and mental health outcomes of breastfeeding for both infants and mothers. The Guideline recommends exclusive breastfeeding until the age of six months, when solid foods are introduced to the infant’s diet. Breastfeeding should be continued until 12 months of age and beyond as complementary feeding if the infant and mother both wish [12]. Many national efforts, including the initial development of the *National Breastfeeding Strategy (1996–2001)*[13] followed by the *Australian National Breastfeeding Strategy 2010–2015*[14], have been initiated to support and promote successful breastfeeding in Australia. With our increasing awareness of the advantages of breastfeeding, health professionals from all disciplines should work together to promote breastfeeding. In Australia, the percentage of women who choose breastfeeding instead of formula-feeding immediately post-partum has increased from approximately 48% in the 1970’s to over 90% in 2010 [15,16]. However, the continuation rate declined sharply with time post birth with percentages of any breastfeeding and exclusive breastfeeding at six months only at 56% and 14% respectively in 2004 [14,17].

A concern for lactating women who are taking medications is the transfer of medicines into breast milk [5,8]. Medicines circulating in the maternal bloodstream can potentially be transferred into human breast milk, exposing breastfed infants to medicines that may potentially be harmful [5,8]. Another concern is the effect of medication on the quantity and quality of breast milk produced, which may impact on the exclusivity, duration and success of breastfeeding [15,16]. Medicines that have been reported to compromise production of milk include cabergoline [18], bromocriptine [19], ergotamine [20], pseudoephedrine [21], and oestrogens [20,22]. Besides conventional medications, some natural substances have also been associated with reduction of breast milk supply. Peppermint, sage and parsley have been used traditionally for weaning, however there is a lack of research-based evidence to support their clinical use [15,23]. While medicine treatments include both conventional and alternative medicines, most available studies have focused on evaluating the transfer of conventional medicines into breast milk.

The use of complementary and alternative medicines (CAMs) is increasingly common worldwide. Research undertaken in the last couple of decades in many countries including the United States [24,25], Canada [26], the United Kingdom [27,28] and the United Arab Emirates [29] all demonstrated substantial increase in the use of CAMs amongst the general population. Research conducted in Australia has shown results consistent with the above findings [30-36]. A prevalence study conducted in 2005 by Xue *et al.*[35] showed that 68.9% of the participants recorded use of one or more forms of CAMs in the previous twelve months of a survey. Zhang *et al.* in 2008 [33] further reported the prevalence and pattern of use of the top 24 most commonly used herbal medicines in Victoria, Australia amongst the general population. These included aloe vera, garlic, green tea, chamomile, echinacea, ginger, cranberry, peppermint, ginseng, ginkgo biloba, evening primrose, dandelion, valerian, liquorice, St. John’s wort, slippery elm, milk thistle, dong quai, black cohosh, bilberry, senna, hawthorn, saw palmetto and chasteberry, in decreasing order of popularity amongst the survey respondents [33].

Many women self-medicate with complementary medicines and supplements, most commonly on recommendation by friends or family, or as prescribed by their health care professionals [37-43]. Studies conducted by Nordeng *et al.*[37] in a group of 400 Norwegian women and by Forster *et al.*[38] among 588 Australian women both showed that 36% of women had taken one or more herbal medicines during pregnancy. We anticipated that some use of herbal medicines was likely to occur during breastfeeding as Stultz *et al.*[44] suggested that women generally use more medications post-partum compared to during pregnancy.

Despite the increasing popularity of herbal medicines, there is currently limited information available on the extent of use and safety of these medicines amongst breastfeeding women. This study aimed to provide current information on the prevalence and pattern of herbal medicines used by women whilst breastfeeding in Western Australia, and to identify commonly used herbal medicines. This information will inform and direct future clinical research. The study also explored the attitudes of breastfeeding women towards herbal medicines and their perceptions of the safety and efficacy of herbal medicines used during breastfeeding, as well as their information-seeking behaviour.

## Methods

This study was conducted using a self-administered structured questionnaire validated through two pilot studies which followed the steps described by Portney and Watkins [45]. The pilot questionnaire was initially circulated among colleagues and lactation consultants, seeking feedback and suggestions. All comments were taken into consideration and the questionnaire was amended following discussion with the research team. The second pilot study was then conducted using the revised questionnaire. The study was approved by the Human Research Ethics Committee of Curtin University.

### Study population and recruitment strategies

The target population was women who were 18 years or older, breastfeeding or who had breastfed in the 12 months prior to the time of the survey. To achieve the study objectives, there were no restrictions as to whether the participant was on any medications or had any medical conditions. Women from all cultural or ethnic backgrounds were eligible for the study. Balancing the need to minimise selection bias and maximise response rate, the decision was made to recruit participants through four main avenues to enable a wide range of participant characteristic types to be recruited:

i) *Mothers and parenting groups.* With written approval from the Australian Breastfeeding Association (ABA), breastfeeding women were recruited from local mothers and parenting groups where the primary investigator (TFS) attended the group meetings.

ii) *Community pharmacies.* A list of 557 WA community pharmacies was provided by the Pharmacy Registration Board of Western Australia. A stratified sampling technique was used to obtain sets of pharmacies within three defined geographical areas: North metropolitan, South metropolitan or regionally based according to postcodes. The lists of pharmacies were arranged in a random order by attaching a computer generated random number to each record and sorting each list by the number. Permission was sought from a total of 30 randomly selected pharmacies, 10 from each region, to place 10 sets of recruitment forms in each pharmacy. The pharmacists in-charge were requested to hand out the sets of forms to any women who visited their pharmacy whom they believed could have been eligible for the study. For example: women who came into the pharmacies with an infant or a young child to purchase any infant-related or breastfeeding-related products, or if they had declared that they were breastfeeding.

iii) *Immunisation clinics and child health centres.* Written site authorisation was obtained from the Child and Adolescent Community Health Executive (CACH), Health Department of Western Australia, to display posters advertising the study at all immunisation clinics and child health centres registered with the CACH in the Perth metropolitan area.

iv) *Advertisement in newspapers and local parenting papers.* This strategy was implemented to advertise the study to the general public.

### Data collection

All participants who had expressed interest in participating in the study were provided, either face-to-face or via postal mail, a set of forms consisting of the participant information sheet, the survey questionnaire and a reply paid envelope. The participant information sheet explained that responses would be treated in confidence in order to guarantee anonymity. Consent was assumed upon return of the completed questionnaire. Participant recruitment and data collection occurred concurrently between February and December 2012.

The questionnaire comprised four sections. See Additional file 1. Sections 1 and 2 collected participants’ demographic profile and their family background characteristics including their origin and ethnicity. This information was collected to explore the association between these factors and the pattern of use of herbal medicines during breastfeeding. Section 3 requested information on the prevalence and pattern of use of herbal medicines during breastfeeding. This section explored the reasons for use, sources of recommendation, users’ perceived efficacy and side effects experienced. Section 4 explored the participants’ information-seeking behaviour as well as their attitudes and beliefs towards the use of herbal medicines during breastfeeding. The types of questions in the questionnaire determined the response options, which were a mix of open-ended and closed-ended questions using Likert-style scaled responses.

### Data analysis and statistics

The survey responses were de-identified and analysed using the Statistical Package for Social Sciences (SPSS) version 20 software for Windows. Qualitative responses obtained from open-ended questions were identified and coded. Reasons for use were categorised and coded concurrently with data entry. Upon completion of data entry, all categories were reviewed and reclassified if necessary to ensure consistency in coding and that there was no duplication. These coded responses were then analysed in the same manner as the closed-ended (quantitative) responses.Quantitative data were summarised using standard descriptive statistics (frequencies and percentages for categorical variables; means and standard deviations for variables measured on a continuous scale). The prevalence of use of herbal medicines during breastfeeding was calculated, along with its 95% confidence interval. Univariate associations between demographic data and use of CAM were assessed using Chi-square statistics or t-tests, as appropriate. A multivariate logistic regression model was used to identify any factors independently associated with use of CAM. The optimum model was obtained using a ‘backwards elimination’ strategy, whereby all demographic variables were initially included in the model, and then dropped, one at a time, until all variables remaining in the model were significantly associated with the use of CAM. Respondents were classed as “users” or “non-users” for the purpose of analysis depending on whether or not they had used any herbal medicines during breastfeeding. A p-value < 0.05 was taken to indicate a statistically significant association in all tests.

## Results

### Respondents

A total of 1118 survey forms were distributed and 304 questionnaires were returned, a response rate of 27.2%. The mean [SD] ages of respondents were 32.8 [4.2] years for users and 32.3 [5.0] years for non-users (p = 0.30). The majority of the respondents resided in the Perth metropolitan area, were born in Australia or New Zealand, had completed secondary school education, had a relatively high total annual household income (≥ AUD 80, 000), had only one child and were not living with their parents. For those respondents not born in Australia, the years spent in Australia was not significantly different (p = 0.39) between users (14.1 [8.9]) and non-users (15.7 [10.8]). The characteristics of respondents and factors affecting the use of herbal medicines during breastfeeding are summarised in Table 1.

**Table 1:** Respondents’ demographic profiles and factors affecting herbal medicines use

**Variable**	**Proportion of users, n/N (%)**	***p*-value**
**Total number of participants**	182/304 (59.9)	
**Residential area, determined by postcode (n = 303)**		0.51
Perth metropolitan	171/284 (60.2)	
Other WA region	10/19 (52.6)	
**Country of birth (n = 304)**		
Australia/New Zealand	97/180 (53.9)	0.0180
Asia/Africa	58/80 (72.5)	
Europe/USA/Canada	27/44 (61.4)	
**Ethnic background (n = 301)**		0.0328
Caucasian	55/105 (52.4)	
Asian	57/80 (71.3)	
Others	68/116 (58.6)	
**Level of education (n = 302)**		0.56
Secondary education	12/24 (50.0)	
Secondary school certificate of education	13/24 (54.2)	
Diploma or advanced diploma	12/16 (75.0)	
Trade certificates I, II, III or IV	15/27 (55.6)	
Bachelor degree	88/137 (64.2)	
Graduate diploma or graduate certificate	19/33 (57.6)	
Master degree	19/31 (61.3)	
Doctoral degree	4/10 (40.0)	
**Total annual household income last year (n = 302)**		0.0219
Low (< AUD 37 K)	7/17 (41.2)	
Middle (AUD 37 K – AUD 80 K)	61/86 (70.9)	
High (AUD 80 K+)	113/199 (56.8)	
**Parity (n = 304)**		0.54
1 child	102/176 (58.0)	
2 children	58/94 (61.7)	
3 children	16/27 (59.3)	
4 children	6/7 (85.7)	
**Living with parents (n = 304)**		0.68
Yes	19/30 (63.3)	
No	163/274 (59.5)	

P-values were obtained from the Chi-square test.

### Prevalence and pattern of use

Participants were classified as ‘users’ if they had specified the use of any herbal medicines for the purpose or intention of treating or managing any medical condition or to improve their health. Amongst the 304 respondents, 182 (59.9%) indicated that they had used one or more herbal preparations for various medicinal purposes during breastfeeding (CI 54.4-65.4%). The number of herbal products used by respondents ranged from none to six, with an average of 1.25 products per participant. Of the 182 respondents who took at least one herbal medicine during breastfeeding, 70 (38.5%) reported use of only one herb, 51 (28.0%) used two herbs, 37 (20.3%) used three herbs, 16 (8.8%) used four herbs, 5 (2.7%) used five herbs, and 3 (1.6%) used six herbs.

Over half (60.4%) of users indicated that the reasons for use of these herbs were breastfeeding-related. Approximately one in four of the respondents (74/304; 24.3%; 95% CI: 19.5% - 29.1%) took one or more herbal medicines specifically to help increase milk production or supply during breastfeeding.

A logistic regression model was used to investigate if any respondent characteristics or demographic factors were associated with the decision to use herbal medicines during breastfeeding, with the results shown in Table 2. The multivariate model shows that respondents with an Asian birthplace were more likely to use herbal medicines, as well as those from middle income families (total annual household income of AUD 37, 000 – AUD 80, 000). These factors were the only two which remained after the backwards-elimination model-fitting strategy. Other variables which were initially included in the model were dropped since they appeared to be not significantly associated with the outcome.

**Table 2:** Logistic regression model: factors associated with the use of herbal medicines

**Variable**	**Odds ratio**	**95% confidence interval**	***p*-value**
**Country of birth**			
Asia	2.20	1.25 to 3.88	0.0061
Other	1 (reference)		
**Family income**			
Low (<$AUD37K)	0.29	0.10 to 0.84	0.0234
Mod ($37 K to $80 K)	1 (reference)		
High (>$80 K)	0.53	0.31 to 0.91	0.0214

A total of 51 different herbal medicines or ingredients were revealed amongst the survey respondents in this study. The top ten most commonly used herbal medicines during breastfeeding in the descending order of popularity were fenugreek (18.4%), ginger (11.8%), dong quai (7.9%), chamomile (7.2%), garlic (6.6%), blessed thistle (5.9%), cranberry (4.9%), fennel (4.9%), aloe vera (3.3%) and peppermint (3.3%). Women were asked to indicate the reasons for use, who recommended the use, and their perceived efficacy of whether the herbal medicine was helpful to address their intended indication. The proportion of women who perceived the herbal medicine as helpful varied from 20.0% to 83.3%. These findings along with their prevalence are shown in Table 3.

**Table 3:** Top ten most commonly used herbal medicines during breastfeeding (in descending order of popularity)

**Common name of herbal medicine**	**Binomial/scientific name**	**n (%) reporting use of this herb**	**n (% of specific herb users) who believed the herb helped**	**Reasons for use (% of specific herb users)**
Fenugreek	*Trigonella foenum-graecum*	56 (18.4)	44 (78.6)	Increase breast milk supply (98.2)
				Boost immune system during cold (1.8)
Ginger	*Zingiber officinale*	36 (11.8)	17 (47.2)	General health enhancement, tradition (65.7)
				Relief of “winds” and “air” (22.9), Others (11.4)
Dong quai	*Angelica sinensis*	24 (7.9)	10 (41.7)	General health enhancement (90.9)
				Others (9.1)
Chamomile	*Matricaria chamomilla*	22 (7.2)	13 (59.1)	Calming and relaxation, stress (86.4)
				Others (13.6)
Garlic	*Allium sativum*	20 (6.6)	7 (35.0)	Boost immune system during cold (38.9)
				General health enhancement, tradition (50.0)
				Antifungal (5.6)
				Improve blood circulation (5.6)
Blessed thistle	*Cnicus benedictus*	18 (5.9)	15 (83.3)	Increase breast milk supply (100.0)
Cranberry	*Vaccinium macrocarpon*	15 (4.9)	6 (40.0)	Urinary tract infections/bladder health (76.9)
				Others (23.1)
Fennel	*Foeniculum vulgare*	15 (4.9)	10 (66.7)	Increase breast milk supply (80.0)
				Relieve of colic (13.3)
				Energy, restore iron levels from blood loss (6.7)
Aloe vera	*Aloe vera*	10 (3.3)	2 (20.0)	Detox (n = 6)
				Aids digestion, intestinal health (n = 1)
				General health enhancement (n = 1)
				Sunburn, cooling effect (n = 1)
Peppermint	*Mentha piperita*	10 (3.3)	7 (70.0)	Calming and relaxation (n = 6)
				Relieve of bloating (n = 2), Others (n = 2)

There were 18 different herbal medicines or ingredients indicated by the respondents specifically as a galactagogue, that is to increase breast milk supply and breastfeeding performance. Table 4 reports on the top seven most commonly used herbal galactagogues along with their perceived efficacy, in descending order of priority.

**Table 4:** Top seven most commonly reported herbal galactagogues

**Common name of herbal galactagogue**	**Binomial/scientific name**	**n (%) reporting use of this herb**	**n (% of specific herb users) who believed the herb helped**
Fenugreek	*Trigonella foenum-graecum*	56 (18.4)	44 (78.6)
Blessed thistle	*Cnicus benedictus*	18 (5.9)	15 (83.3)
Fennel	*Foeniculum vulgare*	15 (4.9)	10 (66.7)
Goat’s rue	*Galega officinalis*	7 (2.3)	7 (100.0)
Nettle/stinging nettle	*Urtica dioica*	5 (1.6)	4 (80.0)
Blackthorn berry	*Prunus spinosa*	5 (1.6)	4 (80.0)
Shatavari	*Asparagus racemosus*	4 (1.3)	4 (100.0)

### Sources of recommendation, supply and information-seeking behaviour

Participants were asked to state who had recommended the use of each of the specified herbal medicines. Responses were tabulated separately, and grouped into seven main categories as presented in Table 5. Approximately two-thirds of the users (n = 112) had chosen to use herbal medicines during breastfeeding based on recommendations from their family members. Prescribers and specialists, including general practitioners, gynaecologists and obstetricians were least likely to recommend use of herbal medicines during breastfeeding, as results have shown that only 2.2% of users were recommended to use herbal medicines by this group of health professionals.

**Table 5:** Sources of recommendation, supply and information

**Survey questions**	n (% of users/respondents)*
***Who has recommended the use of herbal medicines? (n = 182 number of users)***	
Family members	112 (61.5)
Naturopaths, herbal or health food stores	86 (47.3)
Friends	57 (31.3)
Self (including self-reading of magazine, internet)	56 (30.8)
Health professionals (including clinic/child health nurses, midwives, lactation consultants)	26 (14.3)
Pharmacists and pharmacy staff	24 (13.2)
Prescribers and specialists (including doctors/general practitioners, gynaecologists, obstetricians)	4 (2.2)
***Sources of supply: Where have they obtained or purchased their herbal medicines? (n = 182 number of users)***	
Community pharmacies	105 (57.7)
Herbal or health food stores	88 (48.4)
Supermarkets	73 (40.1)
Naturopathic clinics	27 (14.8)
Family or friends	26 (14.3)
Internet	4 (2.2)
***Sources of information: Where to seek information concerning use of herbal medicines during breastfeeding? (N = 303)***	
Pharmacists	154 (50.8)
Doctors	146 (48.2)
Family or friends	139 (45.9)
Internet	133 (43.9)
Lactation consultants	89 (29.4)
Naturopaths or homeopathic practitioners	87 (28.7)
Child health nurses	83 (27.4)
Herbal or health food stores	71 (23.4)
Books, literature or journal articles	59 (19.5)
Others	11 (3.6)

*Does not total 100% as more than one response had been indicated by some participants.

Table 5 also summarises the sources of supply based on the users’ responses. The majority of the users (n = 105) had obtained or purchased their herbal medicines or products from community pharmacies. Health food stores and supermarkets were two other common sources of supply, followed by naturopathic clinics, family and friends and the internet.

All respondents to the survey (users and non-users) were asked to identify resources where they had in the past or would in the future seek information concerning the use of herbal medicines during breastfeeding. Results indicate that respondents were most likely to seek information and advice from pharmacists and doctors. Family and friends as well as internet resources were also common reported sources of information, followed by lactation consultants, naturopaths or homeopathic practitioners, child health nurses, health food stores, and books, literature or journal articles. Despite doctors being identified as one of the common sources of information, only 52 (28.6%) of the users in this study had made their doctors aware of their decision to use herbal medicines whilst breastfeeding.

### Attitudes and beliefs towards the use of herbal medicines during breastfeeding

Over 70% of respondents strongly agreed or agreed that there was a lack of information resources available to them regarding the use of herbal medicines during breastfeeding, whilst 23.6% selected the option “no idea” and 6.3% strongly disagreed or disagreed.

Many of the respondents (43.4%) believed that herbal medicines are generally safer when compared to conventional medicines during breastfeeding. Most (71.6%) had indicated a previous refusal or avoidance of medicine treatments during breastfeeding due to concerns regarding safety of their breastfed infants. When given a choice, the majority of the women (75.9% of respondents) preferred more information to be available regarding the safety and efficacy of herbal medicines specifically when used during breastfeeding.

## Discussion

Although many studies have been conducted to investigate the prevalence and pattern of use of CAMs in Australia in the general population, few have focused specifically on the use of herbal medicines by breastfeeding women. In this study, 59.9% of the women used at least one herbal medicine during breastfeeding for various medicinal purposes. This prevalence (59.9%) appeared to be higher than results from a similar study conducted in a group of Australian women during pregnancy (588 participants; median age 32; 57% born in Australia/New Zealand; 17% born in Asia; 26% born in other countries) published in 2006 [38], which found that herbal medicine use was higher during the postpartum than the prenatal periods [44]. Furthermore, many studies conducted worldwide have shown a steady increase in the use of herbal medicines, most likely due to the increased awareness and availability or accessibility of herbal products [24,29,35-38,43,46]. An association was identified between the respondents’ birthplace or their ethnic background and the decision to use herbal medicines. Women with an Asian background in this study were more likely to use herbal medicines during breastfeeding. A study conducted in Taiwan explored the use of Chinese herbal medicines by women during both pregnancy and postpartum period [46]. The authors not only reported a relatively high prevalence of herbal medicine use in the cohort, but also demonstrated a marked escalation of prevalence from 33.6% during pregnancy to 87.7% during the postpartum period. Our results suggest higher prevalence of herbal medicine use by women from the middle income families, supporting the argument that cost and affordability may be a factor to consider when selecting type of therapy [31,32,35]. The relationship between women from the middle income families and the higher prevalence of herbal medicine use should be further explored.

The most commonly used herbal medicines found in this study were consistent with previously published reports. Herbs which were not used specifically as galactagogues including ginger, dong quai, chamomile, garlic, cranberry, aloe vera and peppermint were all included in the 24 common medicinal herbs used by the general Australian population as reported by Zhang *et al.*[33]. Furthermore, the reported indications for use of these herbs were consistent with the traditional uses in many of the previous studies [33,35,37,38].

Over 24% of respondents took at least one herbal medicine for the purpose of increasing breast milk supply or promoting breastfeeding performance. Amongst the top ten herbs identified in this study, fenugreek, blessed thistle and fennel emerged as the top three herbal galactagogues. Other herbal galactagogues included goat’s rue, nettle, blackthorn berry and shatavari. All these herbs have gained their reputation as galactagogues over the years, however mostly based on anecdotal evidence [43,47,48]. Limited clinical trials or large-scale studies are available to ascertain their efficacy as galactagogues. Nevertheless, this study identified the common herbal galactagogues used by women living in Australia and highlighted the need to conduct clinical research to confirm their efficacy and safety.

### Sources of recommendation, supply and information-seeking behaviour

This study investigated the sources of recommendation and supply, and explored breastfeeding women’s information-seeking behaviour. Family and friends were the most common source of recommendation, yet approximately half of these breastfeeding women (57.7% of reported users) obtained or purchased their herbal medicines from a community pharmacy. This finding indicates a potential role for community pharmacists and pharmacy staff in influencing breastfeeding women’s decisions regarding the use herbal medicines during breastfeeding.

When given a choice, breastfeeding women were most likely to seek information and advice regarding the use of herbal medicines from pharmacists and doctors. Internet resources, family and friends were also commonly reported sources of information. Interestingly, only 52 (28.6%) out of the 182 users of herbal medicines in this study had made their doctor aware of their decision and choice of therapy. Other studies have also revealed a lack of communication between users of CAMs and their doctors in terms of their use of alternative therapies [42,49]. Nevertheless, all health care professionals, including doctors and pharmacists should take the initiative to ask and provide evidence-based advice regarding the appropriateness of using herbal medicines during breastfeeding. Considering the high prevalence of herbal medicines used during breastfeeding and the risk of potential interactions and adverse outcomes, all health care providers, including community pharmacists and pharmacy staff, should routinely ask female customers if they are breastfeeding and if they are using any medicines including CAMs. Although other studies have investigated the role of community pharmacists in providing advice regarding the use of CAMs in the general population [50-55] and the role of community pharmacists in counselling breastfeeding women [56,57], few studies exist to examine the role of this health care professional group in providing advice regarding the use of herbal medicines specifically to breastfeeding women and their families. Besides the community pharmacists, this study has also identified a greater need for both conventional healthcare providers and CAM practitioners to develop an interdisciplinary network, working collaboratively to ensure optimum health outcomes for their clients.

### Attitudes and beliefs

Forster *et al.*[38] suggested the reason for the high prevalence of women not informing their doctors regarding their decision to use herbal medicines during pregnancy was the assumption that CAMs are ‘natural’ and hence safety would not be an issue. It is likely that this factor may also be contributing to the high prevalence of use identified in this study as the majority of the women who participated (43.4%) perceived herbal medicines as safer options compared to conventional medicines during breastfeeding. Although most herbal medicines are readily available over-the-counter without a prescription, it is important to take into consideration the potential risk of drug-disease interactions and interactions between herbal medicines of their choice and medicines prescribed by doctors.

Approximately seventy-percent (70.1%) of the respondents indicated that they either strongly agree or agree that there was a lack of resources available to them regarding the use of herbal medicines during breastfeeding. Nevertheless, some women continued to use their therapy of choice based on limited readily available evidence-based information. Over 70% of respondents indicated that they had previously refused or avoided medicine treatments during breastfeeding due to concerns regarding safety of their breastfed infants. The study has demonstrated the urgent need for further research into this area as both untimely cessation of breastfeeding and mother denial of medicine treatments to meet their medical needs may lead to unwanted consequences.

### Limitations

As the research involved a voluntary self-administered questionnaire, this study may overestimate the use of herbal medicines during breastfeeding as a result of voluntary response bias [45,52]. Women who had a personal interest or were taking herbal medicines may have been more likely to participate in the study. The 2011 Census indicated that 27% of the Australian population were born overseas, with the majority of migrants from European and Asian countries [58]. According to the Australian Bureau of Statistics (ABS), a total of 31, 820 babies were born in Western Australia in 2011 [59]. Assuming approximately 90% of women initiated breastfeeding [16], the sample size of this study is small relative to this population. There was also a low response from women from lower income families and thus the views expressed by the study participants may not accurately reflect those of the complete breastfeeding population. Due to the self-reporting nature of this study, some respondents might not have correctly identified herbal ingredients or could have omitted herbs from the more complex products or formulas. Although the study suggested an association between the use of herbal medicines and users’ country of birth and ethnic background, all questionnaires were administered in English. Further studies conducted in other languages would encourage women from a non-English speaking background to participate, which would also provide an improved representation of the broader population. Despite identifying the potential role of community pharmacists and pharmacy staff, this study did not explore the women’s perspectives and reasons for their choice to utilise community pharmacies. In-depth qualitative studies would be valuable to assess if women’s expectations and needs are met, at the same time identifying areas for improvement in the health care system. All surveys were treated anonymously and hence identification of the recruitment location or avenues of those surveys that were returned was not possible. As the survey included personal information, for the purpose of this study, the researchers felt that it was necessary to maintain anonymity of the participants to ensure privacy and confidentiality.

## Conclusions

The use of herbal medicines is common amongst women during breastfeeding, while information supporting their safety and efficacy is lacking. The presumption of safety for some of these medicines, especially when taken concurrently with other conventional medicines, may not be justified. This Western Australian study provides exploratory data on the use of herbal medicines during breastfeeding and identifies those most frequently used. The results support the need for further research and documentation about the safety of herbal medicines in breastfeeding, allowing breastfeeding women to make informed decisions. The herbal medicines most urgently in need of investigation appear to be fenugreek and ginger. Furthermore the efficacy of fenugreek as a galactagogue requires clinical scrutiny. Health professionals and health care providers should be aware of the latest information regarding safety and efficacy of the commonly used herbal medicines in lactation and provide appropriate advice to breastfeeding women.

Research-based information should be available to breastfeeding women who wish to consider use of all medicines, including herbal or alternative medicines. This could avoid interruption or cessation of breastfeeding due to unnecessary safety concerns, while allowing mothers to receive appropriate pharmacotherapy without compromising breastfeeding performance and the infant’s health. Health professionals have an ethical obligation to continuously improve their professional ability to ensure optimum health outcomes of patients. Hence, the research questions now include whether there is sufficient and reliable information and resources available to health professionals, and if they are confident in advising on the use of herbal medicines during breastfeeding.

## Competing interests

The authors declare that they have no competing interests.

## Authors’ contributions

TFS designed, conducted and analysed the data of the project as part of her PhD degree. LT, JS and LH supervised the project and contributed to the design and analysis of the survey. RP provided statistical assistance and contributed to the analysis of data. All authors read and approved the final manuscript.

## Pre-publication history

The pre-publication history for this paper can be accessed here:

http://www.biomedcentral.com/1472-6882/13/317/prepub

## Supplementary Material

Additional file 1 The study questionnaire. Click here for file
