# Supplementary material for: The use of herbal medicines during breastfeeding: a population-based survey in Western Australia
Source: BMC Complement Altern Med. 2013 Nov 13;13:317. doi: 10.1186/1472-6882-13-317 (PMC3835544; doi:10.1186/1472-6882-13-317)
Supplement: Additional file 1 — The study questionnaire. [file 1472-6882-13-317-S1.pdf]

The Use of Herbal Medicines in Lactation

Curtin University

If you are currently living in Australia, 18 years or older, currently breastfeeding or have breastfed in the past 12 months, we would greatly appreciate your participation in the following survey.

By returning this form, it is assumed that you have given consent to participate and for us to use the data in our research. Please be assured that this survey is completely voluntary and anonymous.
No personal contact details will be recorded. Please refer to the Participant Information Sheet (Approval: PH-03-11).

Section 1: Participant Information

1.1 What is your home postcode? [ ] 1.2 What is your age? [ ]

1.3 What is your country of birth? [ ] 1.4 What is your ethnic background? [ ]

If not born in Australia, how many years have you been residing in Australia? [ ]

1.5 What is the highest level of education you have completed? [ ]

1.6 What is the total annual household income last year?

- ☐ AUD 0 - 6, 000
- ☐ AUD 6, 001 - 37, 000
- ☐ AUD 37, 001 - 80, 000
- ☐ AUD 80, 001 - 180, 000
- ☐ AUD 180, 001 - and over

1.7 How many children do you have? [ ]

(If you are NOT CURRENTLY breastfeeding, please go to Section 2)

Regarding your child currently being breastfed:

1.8 What is his/her age? [ ] Gender: ☐ Male ☐ Female

1.9 Is this your first child? ☐ Yes ☐ No

Section 2: Participant and family background characteristics

2.1 What is your parents' place of origin? [ ]

2.2 What are their ethnic backgrounds? [ ]

2.3 Are you living with your parents or your partner's parents? ☐ Yes ☐ No

2.4 Who were you living with when you were breastfeeding your child? [ ]

2.5 Which of the following best describes your parents or your partner's parents' role in giving advices on decisions regarding your own health or nutrition during breastfeeding?

☐ All the time ☐ Quite frequently ☐ Sometimes ☐ Only very occasionally ☐ Never

2.6 Which of the following best describes your parents or your partner's parents' role in giving advices on decisions regarding your child's health or nutrition?

☐ All the time ☐ Quite frequently ☐ Sometimes ☐ Only very occasionally ☐ Never

Section 3: Use of herbal medicines during breastfeeding

The Therapeutic Goods Regulations 1990[1] defines herbal substances as ***"all or part of a plant or substance (other than a pure chemical or a substance of bacterial origin)"***[1].

3.1 Have you used any herbal supplements or preparations during breastfeeding? ☐ Yes ☐ No

If answered No, please go to Question 3.7.

3.2 Are the reason(s) for using any of them breastfeeding-related? ☐ Yes ☐ No

3.3 Please specify the name(s) of herbal preparations, reason(s) or purpose(s), who recommended and whether it was effective from your personal experience. *(If extra space required, please go to the last page)*

Name(s) of Herb	Why?	Recommended by?	Did it help?

3.4 Have you used any herbal supplements during breastfeeding to help increase milk production or supply during breastfeeding? ☐ Yes ☐ No

If answered Yes, please specify details in the table below. *(If extra space required, please go to the last page)*

Name(s) of Herb	Why?	Recommended by?	Did it help?

3.5 Did you experience any side effect(s) or unwanted effect(s) following the use of herbal supplements during breastfeeding? ☐ Yes ☐ No

If answered Yes, please specify the name(s) of the herb and side effects experienced in the box below.

3.6 Is your doctor aware that you are using these herbal supplements during breastfeeding? ☐ Yes ☐ No

3.7 Have you used any other products, special diets or methods to help increase milk production or supply during breastfeeding? ☐ Yes ☐ No

If answered Yes, please describe the products or methods used in the box below.

Section 4: Sources of herbal medicines and information resources

4.1 Where have you obtained or purchased your herbal products/preparations? Please tick where relevant.

- ☐ Herbal or health food store
- ☐ Naturopathic clinics
- ☐ Pharmacy
- ☐ Supermarket
- ☐ Family or friends
- ☐ Internet
- ☐ Others, please specify ______

4.2 Where have you or would you seek information concerning the use of herbal medicines during breastfeeding? Please tick where relevant.

- ☐ Herbal or health food store
- ☐ Pharmacist
- ☐ Doctor
- ☐ Naturopathic/ Homeopathic practitioner
- ☐ Family or friends
- ☐ Internet
- ☐ Books, literature or journal articles
- ☐ Lactation consultant
- ☐ Child health nurse
- ☐ Others, please specify ______

4.3 Do you agree that currently there is a lack of resources available to you regarding the use of herbal medicines during breastfeeding?

☐ Strongly agree ☐ Agree ☐ No idea ☐ Disagree ☐ Strongly disagree

4.4 Do you agree that using natural herbal medicines is safer than using conventional/prescribed drugs during breastfeeding?

☐ Strongly agree ☐ Agree ☐ No idea ☐ Disagree ☐ Strongly disagree

4.5 In the past, have you refused or avoided any drug treatments during breastfeeding due to concerns regarding safety of your breastfed infant(s)?

☐ Yes ☐ No

4.6 Would you like to know more about the safety and efficacy of herbal medicines use during breastfeeding?

☐ Yes ☐ No

THANK YOU FOR YOUR TIME

References:

1. Therapeutic Goods Regulations 1990 [statute on the Internet]. 1990 [updated 2010 July 1; cited 2010 September 7]. Available from: http://www.comlaw.gov.au/ComLaw/Legislation/LegislativeInstrumentCompilation1.nsf/.

Extra Space for Question 3.3

Name(s) of Herb	Why?	Recommended by?	Did it help?

Extra Space for Question 3.4

Name(s) of Herb	Why?	Recommended by?	Did it help?
